# Increased *in vivo* mitochondrial oxygenation with right ventricular failure induced by pulmonary arterial hypertension: mitochondrial inhibition as driver of cardiac failure?

**DOI:** 10.1186/s12931-015-0178-6

**Published:** 2015-02-03

**Authors:** Gianmarco M Balestra, Egbert G Mik, Otto Eerbeek, Patricia AC Specht, Willem J van der Laarse, Coert J Zuurbier

**Affiliations:** Department of Anesthesiology, Laboratory of Experimental Anesthesiology, Erasmus MC- University Medical Center Rotterdam, Rotterdam, The Netherlands; Department of Anatomy, Embryology and Physiology, AMC, Amsterdam, The Netherlands; Department of Physiology, VUmc Medical Center, Amsterdam, The Netherlands; Department of Anesthesiology, Laboratory of Experimental Intensive Care and Anesthesiology, AMC, Amsterdam, The Netherlands; Department of Anaesthesiology, Academic Medical Centre, University of Amsterdam, Meibergdreef 9, 1105 AZ Amsterdam, The Netherlands

**Keywords:** Oxygen, Mitochondria, Heart hypertrophy, Heart failure, Pulmonary arterial hypertension

## Abstract

**Background:**

The leading cause of mortality due to pulmonary arterial hypertension (PAH) is failure of the cardiac right ventricle. It has long been hypothesized that during the development of chronic cardiac failure the heart becomes energy deprived, possibly due to shortage of oxygen at the level of cardiomyocyte mitochondria. However, direct evaluation of oxygen tension levels within the *in vivo* right ventricle during PAH is currently lacking. Here we directly evaluated this hypothesis by using a recently reported technique of oxygen-dependent quenching of delayed fluorescence of mitochondrial protoprophyrin IX, to determine the distribution of mitochondrial oxygen tension (mitoPO_2_) within the right ventricle (RV) subjected to progressive PAH.

**Methods:**

PAH was induced through a single injection of monocrotaline (MCT). Control (saline-injected), compensated RV hypertrophy (30 mg/kg MCT; MCT30), and RV failure (60 mg/kg MCT; MCT60) rats were compared 4 wk after treatment. The distribution of mitoPO_2_ within the RV was determined in mechanically-ventilated, anaesthetized animals, applying different inspired oxygen (FiO_2_) levels and two increment dosages of dobutamine.

**Results:**

MCT60 resulted in RV failure (increased mortality, weight *loss, increased lung* weight), MCT30 resulted in compensated RV hypertrophy. At 30% or 40% FiO_2_, necessary to obtain physiological arterial PO_2_ in the diseased animals, RV failure rats had significantly *less* mitochondria (15% of total mitochondria) in the 0-20 mmHg mitoPO_2_ range than hypertrophied RV rats (48%) or control rats (54%). Only when oxygen supply was reduced to 21% FiO_2,_ resulting in low arterial PO_2_ for the MCT60 animals, or when oxygen demand increased with high dose dobutamine, the number of failing RV mitochondria with low oxygen became similar to control RV. In addition, metabolic enzyme analysis revealed similar mitochondrial mass, increased glycolytic hexokinase activity following MCT, with increased lactate dehydrogenase activity only in compensated hypertrophied RV.

**Conclusions:**

Our novel observation of *increased* mitochondrial oxygenation suggests down-regulation of *in vivo* mitochondrial oxygen consumption, in the absence of hypoxia, with transition towards right ventricular failure induced by pulmonary arterial hypertension.

## Introduction

Severe pulmonary arterial hypertension (PAH) is associated with poor prognosis. The development of PAH induces hypertrophy of the right ventricle (RV) that often transition into RV failure. Heart failure of the right ventricle during chronic PAH is the most common cause of mortality in severe PAH, with metabolic and energetic derangements as a prominent signature of heart failure [1]. In the hypertrophied and failing heart, the increased systolic wall stress in the ventricular wall would be expected to increase the energy demand, whereas the increased size of the cardiomyocyte and the decreased capillary density is anticipated to diminish the oxygen supply. These observations have led to the suggestion that there is an imbalance between oxygen/energy supply-demand in the hypertrophied heart, which possibly contributes to the transition to the decompensated, failing heart [2]. The often observed decreased PCr/ATP ratio, increased reliance on glucose metabolism and elevated lactate levels [3,4] are in support of such a mismatch, and suggest that the failing heart is an engine out of fuel [5]. One possible cause for reduced ATP production in the failing heart could be the lack of enough oxygen provided to the mitochondria. Hitherto, this question has only been indirectly evaluated through ^1^H NMR techniques which estimate the ratio of deoxygenated to oxygenated myoglobin. Examining several heart failure models using this technique it was concluded that reduced oxygen availability does not play a role in heart failure [3,6,7]. The sensitivity of this technique, however, is such that a signal can only be detected when the entire myocardial tissue investigated reaches an intracellular PO_2_ < 20 mmHg [3]. Since the mean PO_2_ at the level of the mitochondria in intact healthy heart is approximately 35 mmHg [8], the myoglobin technique can only detect large decreases (>40%) in myocardial oxygenation. Thus, relatively smaller decreases or any increase in myocardial oxygenation will go unnoticed with the ^1^H NMR technique. In addition, the mitoPO_2_ within the heart is highly heterogeneous, such that at a mean mitoPO_2_ of 35 mmHg a significant portion of mitochondria may have a mitoPO_2_ < 20 mmHg [8]. These mitochondria may become oxygen-limited, as studies in isolated mitochondria have demonstrated decreased oxygen consumption when PO_2_ drops down in the 10-20 mmHg range [9,10].

Alternatively, ATP production could also diminish due to intrinsic mitochondrial remodelling resulting in suppression of mitochondrial energy production. Suppression of mitochondrial energy metabolism through mitochondrial metabolic reprogamming together with increased glycolysis was recently proposed as intrinsic mechanism in the development of heart failure [11]. In addition, mitochondrial oxygen consumption may also be inhibited through competition of increased levels of NO with O_2_ at the level of cytochrome c oxidase during hypertrophy and heart failure [12]. Irrespective of the precise mechanism, suppression of mitochondrial energy metabolism should be reflected by elevated mitoPO_2_. Therefore, the non-invasive determination of oxygenation within the intact heart may shed light on the important question whether the development of cardiac hypertrophy and the subsequent transition to heart failure is associated with increased hypoxia or suppressed mitochondrial energy production. Since mitochondria are the principal sites of ATP generation, they are the ideal site of determining the prevailing oxygen tension. Our group recently developed a non-invasive technique which allows, for the first time, the quantitative determination of the distribution of mitochondrial PO_2_ within the heart [8,13-15]. Validation studies in several cell lines have demonstrated that the delayed fluorescence signal and its lifetime is determined by mitochondrial PpIX and the amount of mitochondrial oxygen present [15]. Subsequent studies, extending from cells to isolated organs to the *in vivo* organ for liver [14] and heart [8], validated that the lifetime of the specific delayed fluorescence signal measured in all these conditions did indeed reflect the mitochondrial oxygen tension*.* In the current project we now apply this novel technique to examine the *in vivo* mitochondrial oxygenation in the right ventricle during the development of cardiac hypertrophy and failure due to pulmonary arterial hypertension.

## Methods

The experiments of this study were approved by the ethical committee for animal subjects of the Academic Medical Center at the University of Amsterdam and Erasmus Medical Center at the University of Rotterdam. Care and handling of the animals were in accordance with the guidelines for Institutional and Animal Care and Use Committees. Male Wistar rats (n = 22; 7-8 wk, 180-220 g, Harlan, Netherlands) were attributed to either receive an intraperitoneal injection of monocrotaline (MCT) at a dose of 30 mg/kg (n = 7) or 60 mg/kg (n = 9), or normal saline (n = 6). Body weight was monitored for the next 28 days.

### Animal preparation

Invasive measurements were performed on 28 days after intraperitoneal injection. We selected 28 days post MCT administration, because in our hands RV heart failure and sharp reductions in body weight then starts to develop before most of the animals die. Premedication was done with subcutaneous buprenorphine (Temgesic, Schering-Plough, Netherlands; 0.05 mg/kg), 30 minutes before intraperitoneal injection of NaOH-buffered 5-aminolevulinic acid (ALA; 200 mg/kg). ALA was administered to increase mitochondrial PpIX concentration. 30 minutes before instrumentation of the animals a further equal dose of buprenorphine was administered. Anesthesia was induced by isoflurane. 5% isoflurane in oxygen was used for controls, and 3% isoflurane for monocrotaline treated animals (pilot experiments showed increased sensitivity of MCT animals to isoflurane induction). Anesthesia was then similarly maintained for all groups by pentobarbital at 60 mg/kg initial dose followed hourly by 60% of the initial dose. Body temperature was rectally measured and kept at 37 ± 0.5°C with a heating pad. The airway was secured by tracheal intubation following which the animals were ventilated in pressure-controlled mode at 40% oxygen with a positive end-expiratory pressure of 2.5 mbar. The ventilator settings were checked by capnography and blood gases. The carotid artery and jugular vein were cannulated for blood pressure and heart rate monitoring and infusion of fluid (Ringer’s lactate at 10 ml/kg/h) and drugs, respectively. A median upper laparotomy was performed, the diaphragm incised and the pericardium opened. The fiber optic probe for mitoPO_2_ measurements was positioned transdiaphragmally at approximately 1 mm from the right ventricle (RV) of the beating heart.

### Experimental protocol

The measurements of the mitochondrial oxygen partial pressures (mitoPO_2_) were started at 2 hours after ALA injection, to allow sufficient increases in mitochondrial PpIX for the delayed fluorescence measurements. The experimental protocol consisted in the measurement of mitoPO_2_ at varying inspiratory oxygen fraction (40%, 30%, 21%, 40%) and at two dosages of dobutamine (Centrafarm, Etten-Leur, Netherlands), 2.5 μg/kg/min and 5 μg/kg/min. All measurements during the infusion of dobutamine were made at an inspiratory oxygen fraction of 40%. The 30 and 40% FiO2 are necessary to prevent non-physiological low arterial oxygen tension in the MCT animals, probably because in these animals the lung damage impaired ventilation/perfusion matching within the lungs. Every step of the protocol was maintained for 10 minutes before any measurement was performed. At each step 0.2 ml blood was sampled from the cannulated carotid artery and analyzed with a blood gas analyzer (ABL800 flex, Radiometer, Copenhagen). At the end of the experiment, right ventricular pressure was measured by transmyocardial puncture using a 23 gauge needle connected to a pressure transducer and automatic flushing system. After termination of the experiment, heart and lungs were excised and weighed. The heart was subsequently dissected into right and left ventricle, after a small apical part of the heart was removed for histological and biochemical processing. The tissue was immediately frozen in liquid nitrogen and stored at -80°C.

### Mitochondrial PO2 measurements

The principle and setup for *in vivo* mitoPO_2_ measurements was previously reported [8,14,15], and a detailed description of the used setup was recently published [13]. In brief, mitochondrial oxygen tension is measured with the Protoporphyrin IX (PpIX) – Triplet State Lifetime technique. Application of ALA results in the mitochondrial accumulation of PpIX. PpIX displays oxygen-dependent quenching of delayed fluorescence lifetime. Mitochondrial PpIX is excited at 510 nm through an excitation fiber coupled to a tunable laser, and delayed fluorescence at 630-700 nm is detected by an emission fiber connected to a gated microchannel plate photomultiplier. A PC-based data-acquisition system sampled the signal at 1 MHz and averaged 64 laser pulses (repetition rate 20 Hz) prior to analysis. The delayed fluorescence signal was then used to recover the different mitoPO_2_ histograms from fitting the signal by a sum of rectangular distributions of delayed fluorescence lifetimes. Three separate series of 64 pulses were analyzed and averaged at each experimental condition.

### Tissue analysis

Three sections of the apex, 5 μm thick, were cut in a cryostat, collected on slides, fixed in 4% formaldehyde, stained with hematoxylin and eosin, dehydrated and mounted in Entellan (Merck). Images were obtained with a 40x objective, and cross-sectional area of individual myocytes was determined at the level of the nucleus using Image J (imagej.nih.gov/ij/), taking the pixel to aspect ratio into account [16]. Within each section the cross-sectional area was determined for 6-12 cardiomyocytes. RV and LV homogenates were obtained by grinding the frozen tissue into powder in liquid nitrogen, dissolving the powder in 1 ml ice-cold homogenization buffer (0.5% Triton X-100, 250 mM sucrose, 20 mM Hepes (pH 7.4), 10 mM KCl, 1.5 mM MgCl_2_, 1 mM EDTA, 1 mM dithiothreitol, 0.1 mM PMSF, 5 μg/ml leupeptin and aprotinin and 1 μg/ml pepstatin), 15 min incubation, sonication and finally 1 min 10,000 g centrifugation to obtain the supernatant. Citrate synthase activity, as marker of mitochondrial content, and hexokinase and lactate dehydrogenase activities, as markers of glycolysis, were determined in the supernatant according to published spectrophotometric techniques [17,18], and normalised to protein content (Bradford technique).

### Statistics

The data are presented as mean ± SEM. One way analysis was performed followed by Fisher’s probable least-significant-difference post hoc analysis between groups (SPSS Statistics 20). A value of P < 0.05 was considered statistically significant.

## Results

### General characterization of MCT animals

**Figure 1:**
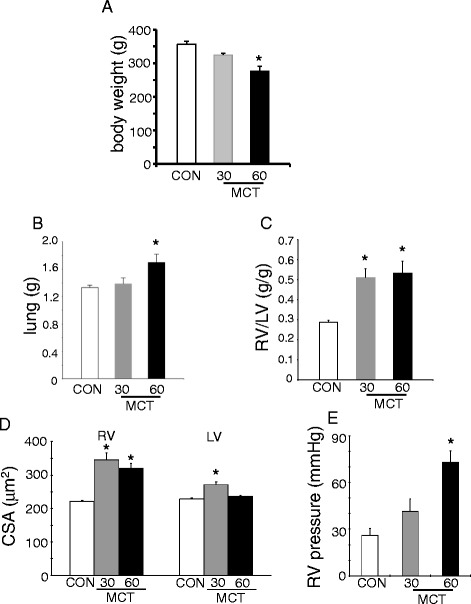
**Body weight and anatomical and functional characteristics of right ventricle of control and MCT treated animals. A**. body weight after 28 d following MCT administration. **B-D**. lung mass, right ventricle (RV) mass relative over left ventricle (LV) mass, and cross sectional area (CSA) of cardiomyocytes in RV and LV, respectively, at end of 28 d following MCT treatment. **E**. systolic RV pressure measured at end experimental protocol (n = 6 per group).

Two 60 mg/kg MCT animals (MCT60) died before 4 wk. One 30 mg/kg (MCT30) and one MCT60 animal died at induction of anesthesia. 6 animals of each MCT group completed the experimental protocol. Body weight developed to a similar degree for all three groups up to 3 wk. Thereafter the MCT60 group started to lose weight, such that at 4 wk body weight was significantly reduced for the MCT60 group as compared to control and MCT30 (Figure 1A). In addition, MCT60 demonstrated increased lung weight at 4 wk (Figure 1B). RV hypertrophy was similar in both MCT groups (Figure 1C and D), whereas peak RV pressure almost tripled in MCT60 rats (Figure 1E). These data indicate compensated RV hypertrophy for the MCT30 group, and the start of right heart failure for the MCT60 group.

### Hemodynamics and blood oxygenation

**Figure 2:**
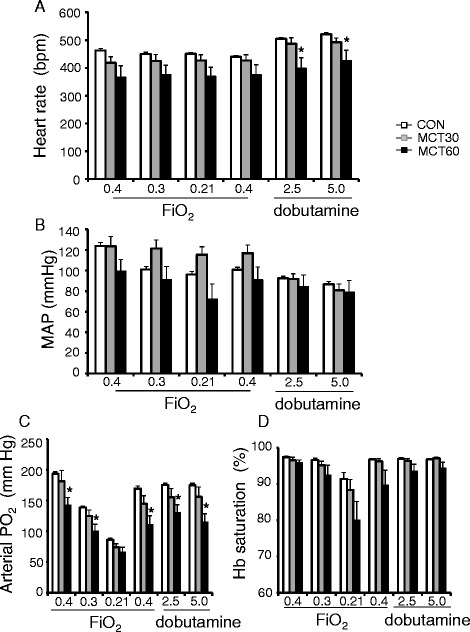
**Hemodynamics (A: heart rate; B: mean arterial pressure (MAP)) and blood oxygenation (C: arterial oxygen tension; D: oxygen saturation of haemoglobin(Hb)) during the changes in inspiration oxygen (FiO**_**2**_ **) and the administration of two dobutamine infusion rates.** * p < 0.05 relative to CON (control). (n = 6 per group).

Physiology of the animals during the experimental protocol is depicted in Figure 2. Although there was a trend that MCT in general lowers hemodynamics in the anesthetized animals, this only reached significance for heart rates (Figure 2A) during the dobutamine infusions; no significant depression of MAP (Figure 2B) was observed following MCT treatment. Reducing FiO_2_ was paralleled with reductions in arterial PO_2_ for both control and MCT animals, whereas arterial PO_2_ was in most conditions significantly higher for control versus MCT animals (Figure 2C). At 30% FiO_2_ the arterial oxygen tension was still at a normal physiological level (~100 mmHg) for the MCT60 group, whereas at 21% FiO_2_ arterial oxygen tension dropped below normal arterial oxygenation levels (<70 mmHg), indicating that at least 30% FiO_2_ was necessary to maintain physiological PaO_2_ in the MCT60 animals. There also was a non-significant trend for decreased oxygen saturation of hemoglobin in MCT animals at 21% FiO_2_ (Figure 2D).

### Mitochondrial oxygen tension

**Figure 3:**
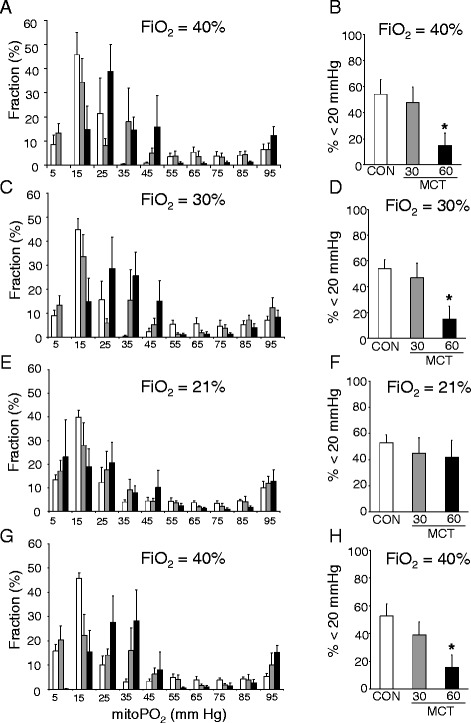
**Right ventricle mitoPO**_**2**_ **measurements during changes in inspiration oxygen content (FiO**_**2**_ **) for control (CON) and monocrotaline-treated animals (MCT). A**, **C**, **E**, and **G**: total distribution of mitoPO_2_ during the different FiO_2_ settings. **B**, **D**, **F**, and **H**: the fraction of mitochondria displaying a mitoPO_2_ < 20 mmHg during the different FiO_2_ settings. * p < 0.05 relative to CON (control). (n = 6 per group).

FiO_2_-dependent PpIX delayed fluorescence signals were obtained *in vivo* from the RV of control and MCT animals and the distribution of mitoPO_2_ histograms determined (Figure 3). At 40% FiO_2_, the distribution of mitoPO_2_ showed a rightward shift towards higher values for the MCT60 RV as compared to control RV (Figure 3A). The fraction of mitochondria with PO_2_ values below 20 mm Hg, assumed to be oxygen limited, was similar between control (54 ± 11%) and the MCT30 group (48 ± 12). However, significant less mitochondria within the RV of the MCT60 group (15 ± 10%) were observed in this low oxygen range as compared to control (Figure 3B). A similar pattern exists when F_i_O_2_ was reduced to 30% oxygen (Figures 3C-D). Only when inspired oxygen supply was further reduced to 21%, the fraction of mitochondria within the 0-20 mmHg PO_2_ range of the RV MCT60 group (42 ± 13%) increased to values similar as control (53 ± 5%) (Figure 3E-F). Resetting F_i_O_2_ again to 40% restored oxygenation to the initial 40% inspiration conditions, indicating reversibility of the phenomenon (Figures 3G-H).

**Figure 4:**
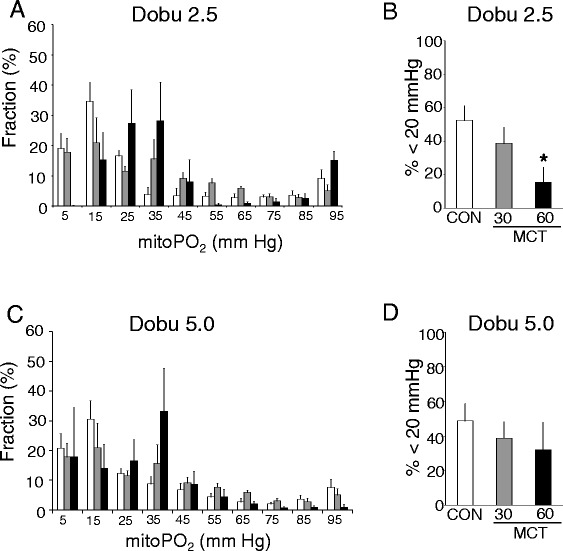
**Right ventricle mitoPO**_**2**_ **measurements during administration of two doses of dobutamine (dobu) for control (CON) and monocrotaline-treated animals (MCT). A**, **C**. total distribution of mitoPO_2_ within RV. **B**, **D**. the fraction of mitochondria displaying a mito PO_2_ < 20 mmHg. *p < 0.05 relative to CON (control). (n = 6 per group)”.

We next analyzed the responses of RV oxygenation towards increases in energy demand by using two different doses of dobutamine infusions (Figure 4). At a dobutamine dose of 2.5 μg/kg/min the differences observed in oxygenation between MCT60 and control RV persisted (Figure 4A-B); only at the highest dobutamine dose did the fraction of mitochondria in the 0-20 mmHg range in the MCT RV (32 ± 16%) become similar to that in control RV (49 ± 10%) (Figure 4C-D).

### Post-experimental metabolic analysis

**Figure 5:**
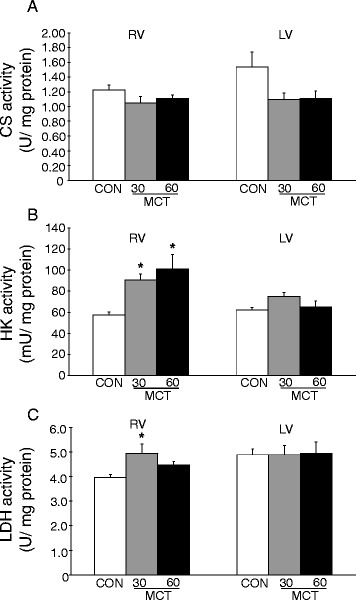
**Metabolic marker enzyme activities in RV and LV of control and MCT treated animals 28 d following MCT administration. A**. citrate synthase (CS) as mitochondrial marker; **B**. hexokinase (HK) as glycolytic marker; **C**. lactate dehydrogenase (LDH), as lactate metabolism marker. *p < 0.05 relative to the respective CON (control). (n = 6 per group).

Finally, we examined metabolic changes within RV and LV at the different stages of pulmonary hypertension (Figure 5). Citrate synthase activity was unaffected by MCT treatment in RV and LV, indicating similar mitochondrial mass among groups. Hexokinase (HK) activity was increased in the RV of both MCT groups. No change in HK activity was observed in LV. In contrast, lactate dehydrogenase (LDH) activity was only increased in the RV of the MCT30 group, but not in the RV of the MCT60 group. LDH activity in LV was unaltered (Figure 5). These data indicated a differential response of glycolytic enzymes to the development of RV failure.

## Discussion

Hypoxia at the level of the cardiomyocyte mitochondria is hypothesized as an important contributing factor in the development of cardiac failure [2,5,19,20]. A critical evaluation of this hypothesis has been missing due to the lack of a technique enabling quantitative measurements of mitochondrial oxygenation within the *in vivo* working heart. In the present study, through application of a recently developed technique by our group, we show that at the initiation of cardiac failure, mitochondrial oxygenation is higher (under baseline conditions) or equal (under decreased oxygen supply or increased work demand conditions) as compared to healthy hearts. No decreased cardiac mitochondrial oxygenation was observed, arguing against hypoxia being an important contributor to the development of right heart failure.

### Mitochondrial oxygenation and cardiac metabolic signature of RV failure

Our observation of less mitochondria with low oxygen in the RV failure developing animals, suggests decreased mitochondrial oxygen consumption in the transition from compensated hypertrophy towards cardiac failure. Decreased oxygen consumption has indeed been directly measured 4-6 wk after MCT administration, although not in intact hearts but in permeabilized cardiac fibers or minced heart tissue [19-21]. Suppression of mitochondrial metabolism together with increased glycolysis was recently proposed as intrinsic mechanism underlying the development of pulmonary arterial hypertension and heart failure [11]. This data would suggest that impairments in energy production (ATP synthesis) in RV heart failure, as reported by different groups [19,22], are mainly metabolically mediated, and not through impairment of oxygen delivery. Theoretically, mitoPO_2_ can also be elevated as a consequence of increases in oxygen supply when going from the hypertrophied to the failing condition. However, such evidence cannot be found in literature. On the contrary, capillary density or coronary blood flow or angiogenesis are commonly decreased in hypertrophy or with the transition towards heart failing [11,16,23]. The increased mitochondrial PO_2_ measured in this study is also not a result from a decrease in mitochondrial capacity, since citrate synthase activities were similar between control and MCT RV. Previous work from our group also demonstrated unaltered mitochondrial capacity in RV of MCT-treated animals [16,24]. We did find elevated HK activities in the hypertrophied and failing RV, consistent with other studies [25-27]. Interestingly, increases in hexokinase were recently also noted as metabolic hallmark of a right heart failure program [28]. The increase in HK is probably an adaptive mechanism, attenuating structural and maladaptive remodelling with pressure-overload [29]. Interestingly, LDH activity was only increased in the compensated hypertrophied RV, but not in the failing RV. Thus, decreased number of mitochondria with low oxygen tension and loss of LDH increase are the specific metabolic markers observed in the present study that sets the developing failing RV apart from the compensated hypertrophied RV. Although increased cardiac HK has been associated with decreased cardiac oxygen consumption [29-31], or decreased LDH may decrease oxygen consumption considering lactate as important substrate for the heart [32,33], further in-depth studies directed at these metabolic hypotheses are needed before any affirmative statements can be made. Alternatively, although not examined in the present study, mitochondrial oxygen consumption may also decrease due to cytochrome c oxidase inhibition by NO, which levels were shown to be increased in congestive heart failure [12,34]. Irrespective of the exact molecular mechanism, our novel observation of higher *in vivo* mitochondrial oxygenation suggests impaired *in vivo* mitochondrial metabolism as a possible trigger for the development of RV failure.

In the current work the chemical substance MCT was used to induce pulmonary hypertension; however, pulmonary hypertension can have several different etiologies (chronic thromboembolic, pre-versus postcapillary pulmonary hypertension, connective tissue disease, etc.). Although we anticipate that, independent of the etiology, any increase in pulmonary arterial pressure will increase RV afterload and thereby most likely start the metabolic, mitochondrial, remodelling phase as described here for the MCT model, further research is warranted to state this more definitively for different pulmonary hypertension etiologies.

### Methodological considerations

Under baseline conditions the animals were mechanically ventilated with 30-40% inspiration oxygen, which is above the ambient air content of oxygen (21%) with spontaneously breathing. The higher inspiration oxygen levels are necessary to compensate for the known ventilation-perfusion defects caused by anesthesia and mechanical ventilation in especially diseased humans and animals [35,36] and are the recommended inspiration oxygen levels with mechanical ventilation used in the clinical arena. Mechanical ventilation in our healthy animals with a FiO_2_ of 21% resulted in an arterial tension of approximately 80 mmHg, below the 100 mmHg normally obtained with spontaneous breathing, indicating that the use of ambient air oxygen levels with mechanical ventilation and anesthesia is indeed associated with insufficient blood oxygenation. In addition, we needed at least a FiO_2_ of 30% to obtain a physiological normal arterial tension of 100 mmHg in the MCT60 animals. Most importantly, even with the *lower* arterial oxygen tension of the MCT60 animals as compared to healthy controls at 30% or 40% FiO_2_, there were still *less* mitochondria with pO_2_ < 20 mmHg in the MCT60 RV, illustrating that the fewer mitochondria with low oxygen are not simple a result of higher arterial oxygen in these sick animals.

The present study used the monocrotaline-induced pulmonary arterial hypertension intervention as a model to study RV hypertrophy and failure. Because almost all animal models are not fully mimicking the clinical features of human pathologies, studies in different models of heart failure, e.g. infarction- or aortic constriction-induced models, will need to be examined to more definitely establish whether increased mitochondrial oxygenation is in general a signature of HF. In addition, although we did not fully characterise RV function with e.g. echocardiography, other studies demonstrated RV dysfunction with 60 mg/kg MCT at 3-6 weeks after MCT administration [11,19,20,37,38]. Together with the reported decreased body weight and increased mortality in the present study it is likely that the MCT60 animals at 4 wk after MCT administration are in the critical transition phase of RV decompensation. However, the use of full RV function characterization with mitoPO_2_ measurements is needed in future studies to describe in more detail the relation between RV dysfunction and RV mitochondrial oxygenation.

## Abbreviations

CS: Citrate synthase
FiO_2_: Inspiration oxygen percentage HK, hexokinase
LDH: Lactate dehydrogenase
LV: Left ventricle
MCT: Monocrotaline
mitoPO_2_: Mitochondrial oxygen tension
PAH: Pulmonary arterial hypertension
PaO_2_: Arterial oxygen tension
PpIX: Protoporphyrin IX
RV: Right ventricle

## References

[1] Vonk-Noordergraaf A, Haddad F, Chin KM, Forfia PR, Kawut SM, Lumens J (2013). Right heart adaptation to pulmonary arterial hypertension: physiology and pathobiology. J Am Coll Cardiol.

[2] Katz AM (1989). Cardiomyopathy of overload: a major determinant of prognosis in congestive heart failure. N Engl J Med.

[3] Murakami Y, Zhang Y, Cho YK, Mansoor AM, Chung JK, Chu C (1999). Myocardial oxygenation during high work states in hearts with postinfarction remodelling. Circulation.

[4] Van Bilsen M, Smeets PJH, Gilde AJ, Van der Vusse GJ (2004). Metabolic remodelling of the failing heart: the cardiac burn-out syndrome?. Cardiovasc Res.

[5] Neubauer S (2007). The failing heart-an engine out of fuel. N Engl J Med.

[6] Bache RJ, Zhang J, Murakami Y, Zhang Y, Cho YK, Merkle H (1999). Myocardial oxygenation at high workloads in hearts with left ventricular hypertrophy. Cardiov Res.

[7] Traverse JH, Melchert P, Pierpont GL, Jones B, Crampton M, Bache RJ (1999). Regulation of myocardial blood flow by oxygen consumption is maintained in the failing heart during exercise. Circ Res.

[8] Mik EG, Ince C, Eerbeek O, Heinen A, Stap J, Hooibrink B (2009). Mitochondrial oxygen tension within the heart. J Mol Cell Cardiol.

[9] Hoffman DL, Salter JD, Brookes PS (2007). Response of mitochondrial reactive oxygen species generation to steady-state oxygen tension: implications for hypoxic cell signalling. Am J Physiol Heart Circ Physiol.

[10] Wilson DF (2013). Regulation of cellular metabolism: programming and maintaining metabolic homeostasis. J Appl Physiol.

[11] Sutendra G, Dromparis P, Paulin R, Zervopoulos S, Haromy A, Nagendran J (2013). A metabolic remodeling in right ventricular hypertrophy is associated with decreased angiogenesis and a transition from a compensated to a decompensated state in pulmonary hypertension. J Mol Med.

[12] Brookes PS, Zhang J, Dai L, Parks DA, Darley-Usmar VM, Anderson PG (2001). Increased sensitivity of mitochondrial respiration to inhibition by nitric oxide in cardiac hypertrophy. J Mol Cell Cardiol.

[13] Harms FA, Voorbeijtel WJ, Bodmer SI, Raat NJ, Mik EG (2013). Cutaneous respirometry by dynamic measurement of mitochondrial oxygen tension for monitoring mitochondrial function *in vivo*. Mitochondrion.

[14] Mik EG, Johannes T, Zuurbier CJ, Heinen A, Houben-Weerts JH, Balestra GM (2008). *In vivo* mitochondrial oxygen tension measured by a delayed fluorescence lifetime technique. Biophys J.

[15] Mik EG, Stap J, Sinaasappel M, Beek JF, Aten JA, van Leeuwen TG (2006). Mitochondrial PO2 measured by delayed fluorescence of endogenous protoporhyrin IX. Nat Methods.

[16] Des Tombe AL, Van Beek-Harmsen BJ, Lee-De Groot MB, Van der Laarse WJ (2002). Calibrated histochemistry applied to oxygen supply and demand in hypertrophied rat myocardium. Microsc Res Tech.

[17] Gürel E, Smeele KM, Eerbeek O, Koeman A, Demirci C, Hollmann MW (2009). Ischemic preconditioning affects hexokinase activity and HKII in different subcellular compartments throughout cardiac ischemia-reperfusion. J Appl Physiol.

[18] Zuurbier CJ, Eerbeek O, Meijer AJ (2005). Ischemic preconditioning, insulin, and morphine all cause hexokinase redistribution. Am J Physiol Heart Circ Physiol.

[19] Daicho T, Yagi T, Abe Y, Ohara M, Marunouchi T, Takeo S (2009). Possible involvement of mitochondrial energy-producing ability in the development of right ventricular failure in monocrotaline-induced pulmonary hypertensive rats. J Pharmacol Sci.

[20] Piao L, Fang YH, Cadete VJJ, Wietholt C, Urboniene D, Toth PT (2010). The inhibition of pyruvate dehydrogenase kinase improves impaired cardiac function and electrical remodeling in two models of right ventricular hypertrophy: resuscitation the hibernating right ventricle. J Mol Cell Cardiol.

[21] Piao L, Marsboom G, Archer SL (2010). Mitochondrial metabolic adaptation in right ventricular hypertrophy and failure. J Mol Med.

[22] Lamberts RR, Caldenhoven E, Lansink M, Witte G, Vaessen RJ, St Cyr JA (2007). Preservation of diastolic function in monocrotaline-induced right ventricular hypertrophy in rats. Am J Physiol Heart Circ Physiol.

[23] Traverse JH, Chen Y, Hou M, Li Y, Bache RJ (2007). Effects of K + ATP channel and adenosine receptor blockade during rest and exercise in congestive heart failure. Circ Res.

[24] Van der Laarse WJ, Des Tombe AL, van Beek-Harmsen BJ, Lee-de Groot MB, Jaspers RT (2005). Krogh’s diffusion coefficient for oxygen in isolated Xenopus skeletal muscle fibers and rat myocardial trabeculae at maximum rates of oxygen consumption. J Appl Physiol.

[25] Do E, Baudet S, Verdys M, Touzeau C, Bailly F, Lucas-heron B (1997). Energy metabolism in normal and hypertrophied right ventricle of the ferret heart. J Mol Cell Cardiol.

[26] Fang YH, Piao L, Hong Z, Toth PT, Marsboom G, Bache-Wig P (2012). Therapeutic inhibition of fatty acid oxidation in right ventricular hypertrophy: exploring Randle’s cycle. J Mol Med.

[27] Zhang WH, Qiu MH, Wang XJ, Sun K, Zheng Y, Jing ZC (2014). Up-regulation of hexokinase 1 in the right ventricle of monocortaline induced pulmonary hypertension. Respir Res.

[28] Drake JI, Bogaard HJ, Mizuno S, Clifton B, Xie B, Gao Y (2011). Molecular signature of a right heart failure program in chronic severe pulmonary hypertension. Am J Respir Cell Mol Biol.

[29] Nederlof R, Eerbeek O, Hollmann MW, Southworth R, Zuurbier CJ (2014). Targeting hexokinase II to mitochondria to modulate energy metabolism and reduce ischemia-reperfusion injury in heart. Brit J Pharmacol.

[30] Nederlof R, Chaoqin X, Gurel E, Koeman A, Hollmann MW, Southworth R (2012). Hexokinase II binding to mitochondria suppress irreversible ischemia reperfusion injury in the beating heart by respiratory inhibition and reduced ROS levels. Circ Res.

[31] Ong SG, Hee Lee W, Theodorou L, Kodo K, Lim SY, Shukla DH (2014). HIF-1 reduces ischaemia-reperfusion injury in the heart by targeting the mitochondrial permeability transition pore. Cardiovasc Res.

[32] Chatham JC (2002). Lactate-the forgotten fuel. J Physiol.

[33] Chatham JC, Gao ZP, Forder JR (1999). Impact of 1 wk of diabetes on the regulation of myocardial carbohydrate and fatty acid oxidation. Am J Physiol Endocrinol Metab.

[34] Traverse JH, Chen Y, Hou M, Bache RJ (2002). Inhibition of NO production increases myocardial blood flow and oxygen consumtion in congestive heart failure. Am J Physiol Heart Circ Physiol.

[35] Nunn JF (1964). Factors influencing the arterial oxygen tension during halothane anaesthesia with spontaneous respiration. Brit J Anaesth.

[36] Nunn JF, Bergman NA, Coleman AJ (1965). Factors influencing the arterial oxygen tension during anaesthesia with artificial ventilation. Brit J Anaesth.

[37] Handoko ML, de Man FS, Happe CM, Schalij I, Musters RJP, Westerhof N (2009). Opposite effects of training rats with stable nd progressive pulmonary hypertension. Circulation.

[38] Korstjens IJM, Rouws CHFC, van der Laarse WJ, van der Zee L, Stienen GJM (2002). Myocardial force development and structural changes associated with monocrotaline induced cardiac hypertrophy and heart failure. J Muscl Res Cell Motil.

